# A Miniaturized Pump Out Method for Characterizing Molecule Interaction with ABC Transporters

**DOI:** 10.3390/ijms20225529

**Published:** 2019-11-06

**Authors:** Emmanuel Sevin, Lucie Dehouck, Romain Versele, Maxime Culot, Fabien Gosselet

**Affiliations:** 1 Laboratoire de la Barrière Hémato-Encéphalique (LBHE), University Artois, EA 2465, F-62300 Lens, France; Emmanuel.sevin@univ-artois.fr (E.S.); Lucie.dehouck@univ-artois.fr (L.D.); romain_versele@ens.univ-artois.fr (R.V.); maxime.culot@univ-artois.fr (M.C.); 2 Blood-brain Barrier Laboratory, Jean Perrin Faculty, Rue Jean Souvraz, 62300 Lens, France

**Keywords:** P-gp, Caco-2, Fluorescence assay, Drug-Drug interactions, screening, drug discovery, ABC transporters

## Abstract

Characterizing interaction of newly synthetized molecules with efflux pumps remains essential to improve their efficacy and safety. Caco-2 cell line cultivated on inserts is widely used for measuring apparent permeability of drugs across biological barriers, and for estimating their interaction with efflux pumps such as P-gp, BCRP and MRPs. However, this method remains time consuming and expensive. In addition, detection method is required for measuring molecule passage across cell monolayer and false results can be generated if drugs concentrations used are too high as demonstrated with quinidine. For this reason, we developed a new protocol based on the use of Caco-2 cell directly seeded on 96- or 384-well plates and the use of fluorescent substrates for efflux pumps. We clearly observed that the new method reduces costs for molecule screening and leads to higher throughput compared to traditional use of Caco-2 cell model. This accelerated model could provide quick feedback regarding the molecule design during the early stage of drug discovery and therefore reduce the number of compounds to be further evaluated using the traditional Caco-2 insert method.

## 1. Introduction

Efflux pumps are a family of protein expressed by several organs such as brain, intestine and testis to protect them from harmful molecules [1,2,3]. Consequently, interactions with these efflux transporters is a major determinant of the pharmacokinetic, safety and efficacy profiles of newly synthetized molecules. Following U.S. Food and Drug Administration (FDA) guidelines, the Caco-2 cell line is widely used in the pharmaceutical industry to identify drug interactions with efflux pumps and therefore to screen for absorption rates of new compounds in the early phases of drug discovery [4]. These cells form a polarized monolayer when seeded on an insert and exhibit an enterocyte-like phenotype. They also display several characteristics associated with the physical and metabolic barrier of the intestinal epithelium [5] including the expression of several efflux transporters, notably ABCB1 (aka P-gp), ABCG2 (aka BCRP) or ABCCs (aka MRPs) members [6]. Assays performed to predict interaction of molecules with efflux pumps consist to generate and calculate an efflux ratio (B/A ratio) of the molecule, i.e., the ratio between the apparent permeability coefficient (Papp) value from the basolateral-to-apical transport across the polarized Caco-2 monolayer (Papp, b-to-a) divided by the Papp value for the apical-to-basolateral transport (Papp, a-to-b).

However, previous works have showed limitations of the efflux ratio method. First, the time and high cost of drug quantification by mass spectrometry reduce the number of test compounds and represent shortcoming for performing this assay in a throughput way. Secondly, compounds with high passive diffusion or low permeability may result in false negatives [7]. Therefore, the compound transport will be limited due to the diffusion rate across basolateral membrane and, therefore, the B/A ratio may be underestimated. At least, another major practical shortcoming of this assay is the long culturing period of at least 21 days to allow for differentiation of the Caco-2 cell monolayers [8]. This long culturing period limited the throughput and usefulness of the model but we successfully reduced this culturing period at 6 days [5].

Therefore, we designed this study to improve the use of Caco-2 cells in the early steps of drug discovery and to reduce the cost of this kind of experiment. Sixteen drugs have been selected based on their ability to interact or not with these efflux pumps [7,9,10]. Method development was achieved by culturing Caco-2 cells directly on collagen-coated 96-well plates for a minimum of 6 days and then by loading the cells with specific fluorescent probe for P-gp, BCRP and MRPs. The cells were then exposed to known P-gp, BCRP or MRPs substrates, non-substrates, test compounds and inhibitors. Efflux of the probes was monitored by tracking the fluorescence eliminated from the cell with a fluorescent plate microreader. The real-time observed fluorescence values correlate with efflux activity of P-gp, BCRP and MRPs. Then, in a second step, we miniaturized this system into a 384-well format, allowing to considerably gain time and money when screening several molecules interaction with efflux pumps. Altogether, our results confirm that we developed a rapid, higher throughput, cost-effective Caco-2 screening method for the compounds and endogenous molecules that interact with different and major efflux protein such as P-gp, BCRP and MRPs. This method might be used in complement of the efflux ratio experiment in the early stage of new molecular entity characterization in drug development.

## 2. Results

### 2.1. Caco-2 Cell Culture

**Figure 1:**
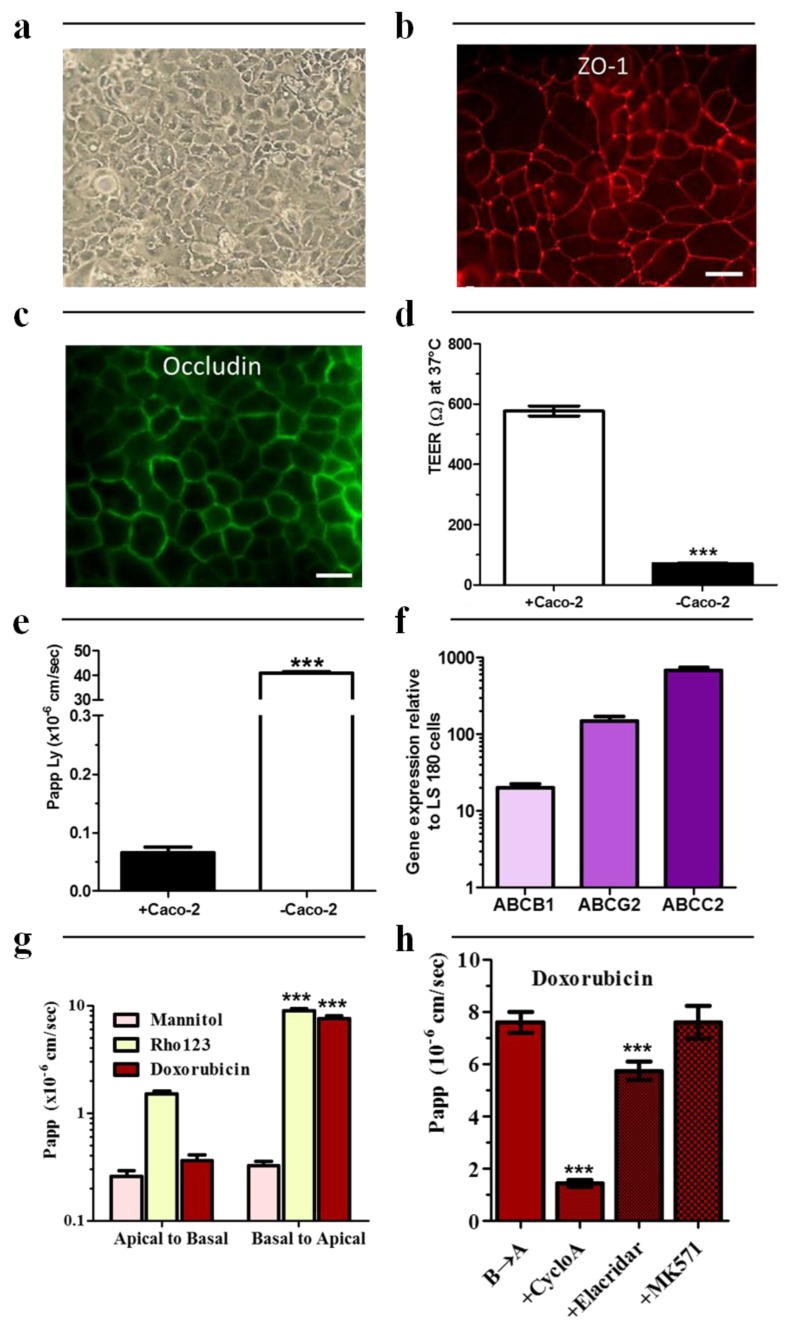
Caco-2 cells were cultivated with the traditional 21 days protocol 24-well plate inserts. (**a**) Morphological characteristics of Caco-2 cells. (**b**) Fluorescent labelling of associated tight junction protein Zonula Occludens-1 (ZO-1) using immunostaining technique. (**c**) Fluorescent labelling of tight junction protein Occludin. (**d**) TEER measurement (*n* = 3). (**e**) Apical-to-basal (a→b) apparent permeability to Lucifer yellow (100 µM) (n = 3). (**f**) Relative expression of *ABCB1*, *ABCG2* and *ABCC2* mRNA measured using real-time quantitative PCR. β-actin was selected as an endogenous mRNA to normalize for differences in the amount of total RNA. (**g**) a→b and b→a apparent permeability to ^14^C-mannitol (37 × 10^3^ Bq.ml^−1^), R123 (20 µM) and doxorubicin (20 µM). (**h**) b→a apparent permeability to doxorubicin (20 µM) with and without cyclosporin A (10 µM), elacridar (0.5 µM) or MK571 (30 µM). Data are represented as means ± s.e.m. *** *p* < 0.001. (b) and (c), Scale bars = 50 µm.

Because culture conditions and cell origin can deeply affect Caco-2 morphology and behavior [4,11,12], we first demonstrated that our Caco-2 cultures form compact, homogenous monolayer of mostly small diameter cells (Figure 1a).

Although no modification of morphology was observed with the increase in passage number (not shown), we only use Caco-2 cells during only ten passages in order to retain the same phenotype as suggested previously [4]. The Caco-2 cells showed a continuous and unique expression of the associated tight junction protein ZO-1 and tight junction protein Occludin at cell–cell contacts (Figure 1b,c respectively). The absence of ZO-1 in the nucleus demonstrates the maturation state of cells and the absence of remodeling at cell–cell contacts. As Mycoplasma is a frequent contaminant of cell cultures and because this prokaryotic organism can modify many aspects of genetic and physiology of cells, including cell growth, metabolism, morphology and attachment [13,14], we validated the absence of any contamination in cell cultures using two different methods. The first one consists in directly staining nuclei of Caco-2. As shown in Figure S2a, no DNA from mycoplasma was observed in the cytoplasm of our Caco-2 cell cultures when compared with a positive control, i.e., contaminated cells (Figure S2b). The absence of mycoplasma contamination in our Caco-2 cell cultures was also confirmed using a commercial mycoplasma detection kit from Lonza™. Caco-2 cultures gave ratio values very close to the negative control, 0.58 ± 0.02 and 0.68 ± 0.15, respectively. Much higher values were obtained for contaminated Caco-2 cell line and positive control, 6.25 ± 1.32 and 72.59 ± 5.40, respectively (Figure S2c).

Then, barrier functions of Caco-2 cells were determined with two different methods: apparent permeability to hydrophilic compound (Papp calculation) and transepithelial electrical resistance (TEER) measuring the resistance to passive ion transport (Figure 1d,e respectively). The Papp of Lucifer yellow was 0.066 ± 0.016 × 10^−6^ cm/s (*n* = 3). This low permeability value clearly demonstrates that the Caco-2 cell line displays the barrier properties required to investigate drug passage across physiological barriers [4]. The TEER value obtained was 576.70 ± 16.65 Ω (*n* = 3). Again, this result is similar to these obtained in the literature [15,16]. 

Efflux transport system was then evaluated in our culture conditions. Expression of P-gp, BCRP and MRP2 (gene names *ABCB1*, *ABCG2* and *ABCC2*, respectively) were quantified by real-time quantitative PCR (Figure 1f). The relative concentration (mRNA target/β-*ACTIN*) was 4.35 ± 1.5 × 10^−4^ mRNA for *ABCB1*, 53.30 ± 9.13 × 10^−4^ mRNA for *ABCG2* and 257.90 ± 21.10 × 10^−4^ mRNA for *ABCC2*, respectively. In addition, the relationships mRNA expression—transporter activity were determined by investigating the permeability of known efflux transporter substrates (Figure 1g). a→b (absorptive) and b→a (secretory) permeability of mannitol (negative control), rhodamine 123 (R123, P-gp, and BCRP substrate [17]) and doxorubicin (P-gp and MRPs substrate [18]) were determined. As expected, the apparent permeabilities in absorptive direction were low: 0.26 ± 0.03 × 10^−6^ cm/s, 1.51 ± 0.01 × 10^−6^ cm/s and 0.36 ± 0.03 × 10^−6^ cm/s for mannitol, R123 and doxorubicin, respectively. The apparent permeabilities in secretory direction were upper than in absorptive, 8.94 ± 0.40 × 10^−6^ cm/s and 7.60 ± 0.40 × 10^−6^ cm/s for R123 and doxorubicin but similar for mannitol (i.e., 0.32 ± 0.03 × 10^−6^ cm/s) suggesting a secretory active transport for R123 (efflux ratio (ER) = 5.96 ± 0.26) and doxorubicin (ER = 21.85 ± 1.11). In addition, in the presence of the multidrug resistance reversal agent cyclosporin A or elacridar (Figure 1h), the b→a permeability of doxorubicin was reduced by 80% and 25%, respectively, demonstrating an active efflux pump activity in these cells.

### 2.2. Concentration-Dependent Permeability of Quinidine to the Caco-2

**Figure 2:**
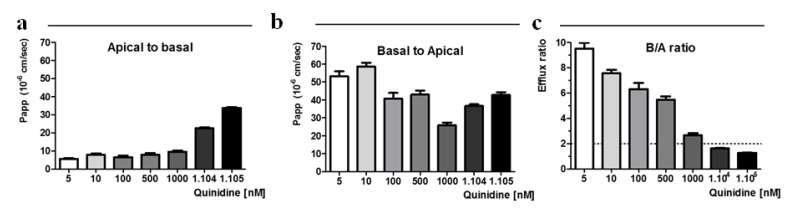
Effect of donor concentration on the transport of ^3^H-Quinidine in Caco-2 cells. Apparent permeability was measured in two directions, (**a**) absorptive apical-to-basal (a→b) and (**b**) secretory basal-to-apical (b→a) at various donor concentrations (0.005–100µM). (**c**) Permeability ratio of quinidine was calculated. Data are represented as means ± s.e.m (*n* = 3).

To demonstrate the limitation of ER calculation in transport assay designed to screen efflux pump substrates or inhibitors, we performed the absorptive (Papp a→b) and secretory (Papp b→a) permeabilities of quinidine, a very well known P-gp substrate and inhibitor [19], at various donor concentrations (0.005 to 100 µM) (Figure 2). 

At the lower concentration range (below 1 µM), a→b permeabilities of quinidine were low and constant, between 5.61 ± 0.76 × 10^−6^ cm/s and 7.87 ± 1.62 × 10^−6^ cm/s for 5 and 500 nM respectively. At the higher concentrations, (10 µM and 100 µM) the permeability values increased strongly to reach 33.76 ± 0.73 × 10^−6^ cm/s at 100 µM. The b→a apparent permeabilities of quinidine were highest at the lower concentrations and reached rapidly a value around 40 × 10^−6^ cm/s from 100 nM. As shown in Figure 2c, the calculated efflux ratio (B/A ratio) of quinidine decreased with increased donor concentration and became approximately a unit at 10 µM and more, suggesting saturation of P-gp-mediated efflux. Therefore, for quinidine concentration above 1 μM, the efflux ratio assay does not discriminate quinidine as a P-gp substrate clearly demonstrating the limitations of the ER calculation method in Caco-2 cells model.

### 2.3. Drug Characterization: Caco-2 Pump Out Assay

**Figure 3:**
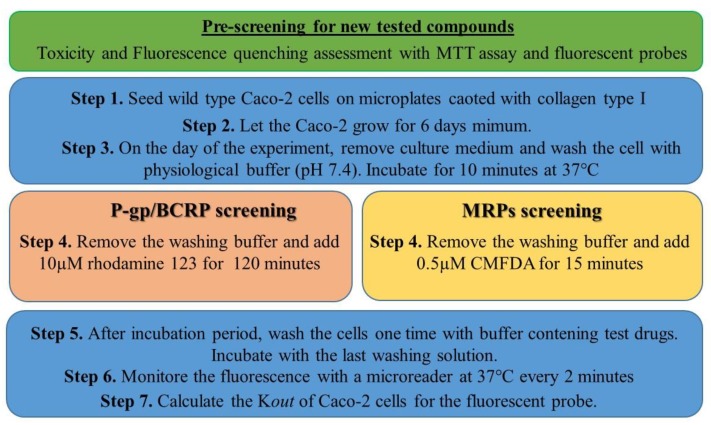
Protocol for the P-gp/BCRP and MRPs microplate screening method.

Therefore, we developed a new method in order to counteract this limitation of the use of Caco-2 cells seeded on an insert. The newly proposed functional assay is based on the capacity of Caco-2 cells to pump out substrates of efflux pumps P-gp, BCRP and MRPs from intracellular cell compartment or cell membrane (summarized in Figure 3). In this assay, R123, is used as P-gp and BCRP substrate at a final concentration of 10 μM. 5-chloromethylfluorescein diacetate (CMFDA), final concentration 0.5 µM, is used as MRPs substrate [20]. 

These two fluorescent probes are then added in washing buffer without preincubation with modulators or substrates. After 15 or 120 min incubation period for CMFDA or R123, respectively, the cells were washed once with RH buffer with modulators or substrates, thereafter the fluorescence is measured immediately during one hour. 

**Figure 4:**
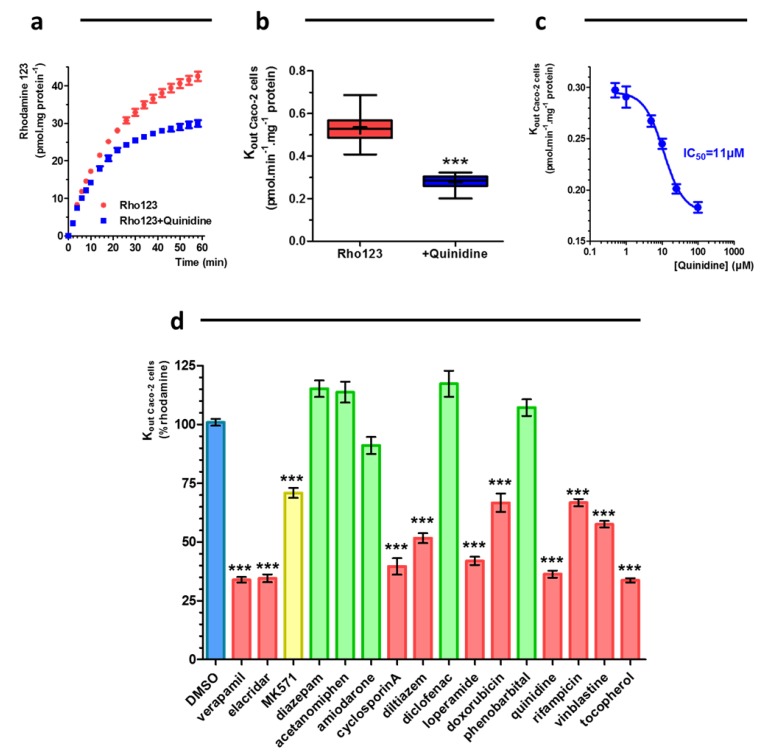
(**a**) Excretion of rhodamine 123 after incubation of cell Caco-2 monolayers in absence or in presence of 100 µM quinidine (mean ± s.e.m., *n* = 12). (**b**) Rate of excretion of rhodamine 123 after an incubation of cell Caco-2 monolayers in absence or in presence of 100 µM quinidine (box-and-whisker diagram, whiskers: min to max, mean: cross, *n* = 12, *** *p* < 0.001). (**c**) Concentration-dependent effects of quinidine on R123 excretion from Caco-2 cells (mean ± s.e.m., *n* = 12). (**d**) Effect of drugs on Rate of excretion of rhodamine 123 (mean ± s.e.m *n* = 12–66, *** *p* < 0.001).

**Figure 5:**
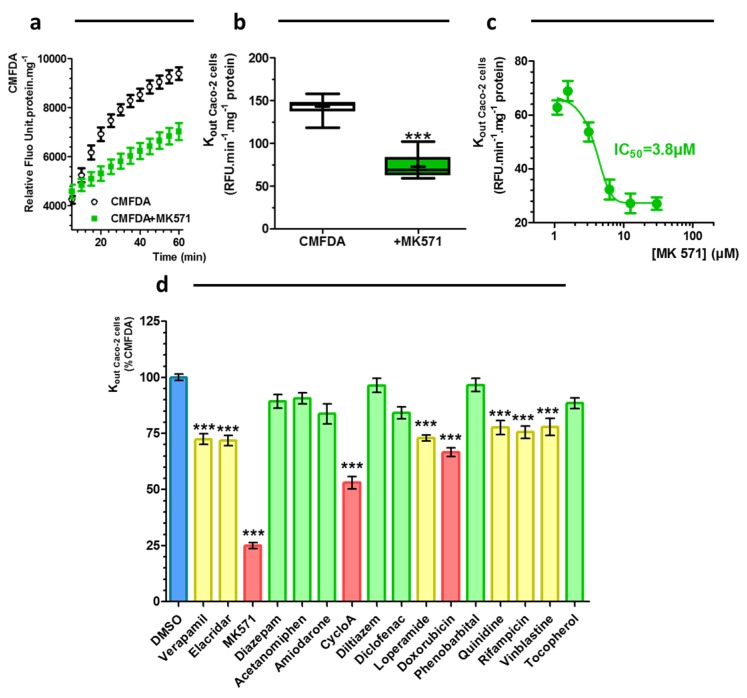
(**a**) Excretion of CMFDA after incubation of cell Caco-2 monolayers in absence or in presence of 50 µM MK571, (mean ± s.e.m, *n* = 12). (**b**) Rate of excretion of CMFDA after an incubation of cell Caco-2 monolayers in absence or in presence of 50 µM MK571 box-and-whisker diagram, whiskers: min to max, mean: cross, *n* = 12, *** *p* < 0.001). (**c**) Concentration-dependent effects of MK571 on CMFDA excretion from Caco-2 cells (mean ± s.e.m., *n* = 12). (**d**) Effect of drugs on Rate of excretion of CMFDA (mean ± s.e.m, n = 12–54, *** *p* < 0.001).

Time-dependent excretion was observed for both R123 and CMFDA (Figure 4a et Figure 5a, respectively). The expelled amount of fluorescence is linear in the time range between 10 to 30 min. When the efflux were studied in presence of 100 µM of quinidine or 50 µM (3-[[3-[2-(7-chloroquinolin-2-yl)vinyl]phenyl]-(2-dimethylcarbamoylethylsulfanyl) methylsulfanyl] propionic acid (MK571), the rates of excretion of the fluorescent probes were reduced. The K_out_ of R123 or K_out_ of CMFDA were reduced by 48% (*n* = 12) or by 49% (*n* = 12) in the presence of the P-gp substrate (Quinidine 100 µM, Figure 4b) or MRP inhibitor (MK571 50 µM, Figure 5b), respectively. 

Concentration-dependent effects of quinidine and MK571 were also evaluated. As shown in Figure 4c and Figure 5c, these effects were concentration dependent. IC50 values towards P-gp/BCRP activity or MRPs activity were determined for quinidine and MK571 by non-linear regression of pump out assay data. 

These IC50 are 11 µM for quinidine and 3.8 µM for MK571. For quinidine, P-gp IC50 is comparable to IC50 values based on the digoxin ER, i.e., 4.9 ± 0.90 µM [3]. For MK571, MRPs IC50 is also comparable to the published values IC50 = 2.43 µM [21]. The usefulness of the assay was studied by determining the interaction of a series of drugs with potential interaction with P-gp/BCRP and/or MRPs. All drugs were tested at 50 µM excepted cyclosporin A, elacridar and D-α-Tocopherol polyethylene glycol 1000 succinate (10 µM) and doxorubicin (20 µM) according to the absence of toxicity on Caco-2 cells obtained with a MTT cytotoxity test (Figure S3). Putative errors of fluorescence quantification were also evaluated with quenching tests and a limit of quantification of fluorescent probes was evaluated in presence of all test compounds (Figure S1a,b, for R123 and CMFDA, respectively). None of them showed any quenching effect on both fluorescent probes. Cyclosporin A [22], diltiazem [7], doxorubicin [7], D-α-Tocopherol polyethylene glycol 1000 succinate [23], elacridar [7], loperamide [7], quinidine [7], rifampicin [7], verapamil [7] and vinblastine [7], all previously described as being P-gp/BCRP modulators (transported substrates and non-transported inhibitors) significantly decreased K_out_ of R123 by more than 30% (p < 0.001) (Figure 4d and Table 1). Interestingly, a slight reduction of rate out of cell of R123 was observed in the presence of MK571 (p < 0.001). MK571 is known, as numerous active drugs, as elacridar, Ko143, loperamide, thioridazine to have an overlapping inhibitory specificity. Concentration dependent inhibitory effect (IC50) of MK571 was previously determined at 26, 50 and 10 µM for P-gp, BCRP and MRPs respectively [24].

For MRPs-drug interaction assessment, MK571, cyclosporine A and doxorubicin decreased strongly the rate out of CMFDA (Figure 5d). Elacridar, loperamide, quinidine, rifampicin, verapamil and vinblastine reduced by more than 20% the k_out_ of CMFDA (p < 0.001). No effect for other molecules were observed.

Altogether, these data demonstrate that our fast and cheap system, without discriminating between efflux pumps substrates or inhibitors, might be used for screening drug interaction with efflux pumps such as P-gp, BCRP and MRPs.

### 2.4. Miniaturization of the Caco-2 Pump Out Assay

**Figure 6:**
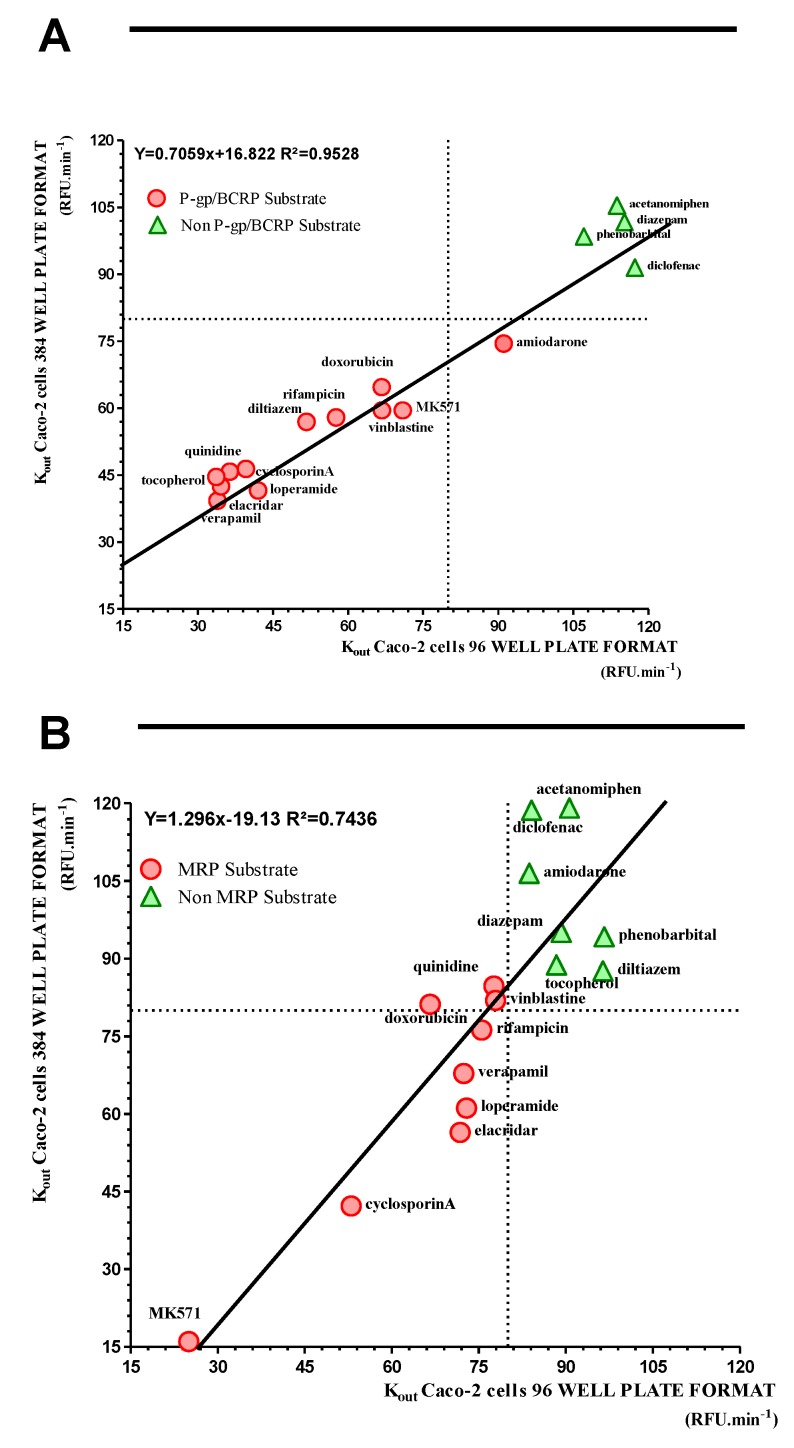
(**a**) Correlation between K_out_ of R123 obtained using the 96- and 384-well plate formats. (**b**) Correlation between K_out_ of CMFDA obtained using the 96- and 384-well plate formats. Classification: Interaction with efflux pumps when K_out_ ≤ 80% of control.

We then decided to miniaturize this system to rather use 384-well format instead of the 96-well format. All the data are summarized in Table 1. Figure 6a,b show that very good correlations are obtained when we compare the drug interaction with P-gp/BCRP (R² = 0.9528) and MRPs (R² = 0.7436) in the two formats. These correlations were confirmed by the value of the mean of ratio K_out_ obtained between the two tested culture formats. Indeed, these values are very close to the unit, i.e., ratio k_out_ for P-gp/BCRP and MRPs are 1.009 ± 0.163 and 1.002 ± 0.230, respectively. Thus, decreasing the number of assays per plates will allow for sure to increase the speed of the screening and to decrease the cost of this kind of experiments.

## 3. Material and Methods

All cell culture reagents and media were obtained from Gibco (Life Technology, SAS Saint Aubin, France), except trypsin-EDTA solution (BiochromAG, Berlin, Germany). All flasks were obtained from Corning (New York, USA). The Transwell polycarbonate HTS 24-well plate inserts (surface area: 0.33 cm²–0.4 μm pore size) were obtained from Costar (Corning Incorporated, NY, USA). ^14^C-mannitol (Mannitol D-[1-14C]) was obtained from Perkin Elmer, ^3^H-quinidine was purchased from BioTrend. Acetanomiphen, amiodarone, cyclosporin A, diazepam, diclofenac, diltiazem, doxorubicin, D-α-Tocopherol polyethylene glycol 1000 succinate, elacridar, loperamide, lucifer yellow, MK571, phenobarbital, quinidine, rhodamine 123, rifampicin, verapamil and vinblastine were obtained from sigma–Aldrich (Saint Quentin Fallavier, France). MTT ((3-(4,5-dimethylthiazol-2-yl)-2,5-diphenyltetrazolium bromide) tetrazolium reduction assay) Cell Proliferation and Cytotoxicity Assay Kit was obtained from Alphabioregen (Boston, Massachusetts, USA) and Green 5-chloromethylfluorescein diacetate (CMFDA) Dye from Thermofisher scientific (Waltham, Massachusetts, USA).

### 3.1. Cell Culture

0.4 × 10^5^ Caco-2 cells were seeded on 25 cm^2^ plastic flask and changed every second day with complete medium containing Dulbecco′s Modified Eagle′s Medium (DMEM) high glucose (4500 mg/L) with L-glutamine (584 mg/L) supplemented by 10% of Fetal Calf/Bovine Serum, 1% of non-essential amino acids without L-glutamine and 1% of penicillin and streptomycin solution. Caco-2 cells were trypsinized after 3 days of incubation while they cover 80%–90% of the flask and seeded at a density of 5 × 10^5^ in 75 cm^2^ flask in complete medium. After 5 to 6 days, Caco-2 cells reach high cells density (>0.5 × 10^5^ cells/cm^2^) and are then split into rat tail Collagen type I 96-well plates, 384-well plates or in HTS 24-well plates with 0.4 μm polycarbonate membrane inserts. Collagen type I 96-well or 384-well plates were seeded at 30 × 10^3^ cells/cm² and cultivated during 6 days. HTS 24-well plates with membrane inserts were seeded at 200 × 10^3^ cells/cm² and cultivated during 21 days according with the standard procedure. Media was renewed every second day.

### 3.2. Mycoplasma Detection

MycoAlert from Lonza was used for the mycoplasma detection assay in Caco-2 culture. Supernatants of culture were mixed with MycoAlert reagents according to the manufacturer instruction and the luminescence was measured after 10 min using microplate fluorimeter (BioTek, H1, Vermont, Winooski).

### 3.3. Caco-2 Pump Out Assay

After a cell culture period of 6 days, Caco-2 cells microplates were washed once with HEPES-buffered Ringer′s (RH) solution (NaCl 150 mM, KCl 5.2 mM, CaCl_2_ 2.2 mM, MgCl_2_ 0.2 mM, NaHCO_3_ 6 mM, Glucose 2.8 mM, HEPES 5 mM, water for injection), pH = 7.4 and incubated for 120 min with 10 µM rhodamine 123 (R123) or for 15 min with 0.5 µM 5-chloromethylfluorescein diacetate (CMFDA). After incubation period, Caco-2 cells were washed 2 times with RH buffer with or without test compounds. The rate out (K_out_) of Caco-2 cells for fluorescent dyes were monitored by fluorescence measuring (λex = 501 nm and λem = 538 nm for R123, and λex = 485 nm and λem = 538 nm for CMFDA) using a microplate fluorescence reader (BioTek, H1, Vermont, Winooski). The amount of dye expelled from the cells was measured at 37 °C and every 2 min during one hour. The rate out of cells of dye was calculated as the slope of the curve of the cumulative amount of dye against the time (K_out_ = ΔpmolΔt) and compared with the value in the presence of test compounds. Verapamil and MK571 were used as positive controls (Substrates or inhibitors) for P-gp [25] and MRPs efflux [22] pumps, respectively. Elacridar was used as inhibitor of P-gp and BCRP [26].

### 3.4. TEER Measurements

TEER (in Ohm) of Caco-2 cells cultured in 24-well plates with 0.4 μm polycarbonate membrane inserts (Costar) was measured using the Millicell-ERS (Electrical Resistance System, Millipore Corporation, Burlington, Massachusetts, USA).

### 3.5. mRNA Extraction and RT qPCR

After 2 washes with cold RH buffer, Caco-2 cells were lysed with RLT lysis buffer (Quiagen, Courtaboeuf, France). mRNA extraction was then performed using commercially available RNeasy mini kit (Qiagen, Courtaboeuf, France) in accordance with manufacturer’s instructions. The amount of RNA and the purity were measured via spectrophotometer (absorbance at A260 nm and A280 nm). Reverse transcription (RT) was performed in a final volume of 20 µL containing 250 ng of RNA, RT-MIX (BioRad iScriptTM Reverse Transcription Supermix) and H_2_O. The samples were incubated in Thermal Cycler (MJ Research, Hampton, NH, USA) using the following protocol: priming 5 min at 25 °C, reverse transcription 30 min at 42 °C and heating to 85 °C for 5 min to inactivated the reverse transcriptase. Expression levels of transporters studied were evaluated by real-time PCR (qPCR). The templates obtained by the RT were used for qPCR amplifications employing CFX96 Real Time System (Biorad), the Supermix (SsoFastTM EvaGreen Supermix, BioRad) and specific primers listed in Table 2. Specificity and efficacy of each primer was tested before their use. The experimental cycling step condition consisting in an initial enzyme activation at 95 °C for 30”, 39 cycles of denaturation at 95 °C for 5” and annealing/extension step at 60 °C for 5”. β-actin was used as housekeeping gene to normalize mRNA.

### 3.6. Transport Studies

Drug solutions were prepared in RH buffer at a final concentration of 100 μM for lucifer yellow, 5 µM for R123 and doxorubicin, 37 KBq.mL^−1^ for ^14^C-mannitol and 0.005 µM to 100 µM for ^3^H-quinidine. For a→b transport experiment, 0.2 mL of the drug solution was placed on the apical side of the cells and samples were taken from the basolateral compartment. For b→a transport experiment 0.8 mL of the solution was placed on the basolateral side of the cells and samples were taken from apical side. Cells were equilibrated for 20 min in RH buffer prior to the transport experiment, and then incubations with compounds were performed at 37 °C under agitation. After one hour, aliquots were taken from each compartment. The amount of radio-labelled compounds ^14^C-Mannitol, ^3^H-quinidine and fluorescent compounds R123, doxorubicin, lucifer yellow was determined using liquid scintillation analyzer, TRI-CARB 2100 (Packard Instrument Company, Meriden, USA) and fluorescence spectrophotometry (BioTek, H1, Vermont, Winooski), respectively. The apparent permeability coefficient (Papp in cm/s) was determined according to the equation: Papp = J/AC0 where J is the rate of appearance of the drug in the receiver chamber, C0 is the initial concentration of the solute in the donor chamber and A, the surface area of the filter [5].

### 3.7. Immunostaining

Cells were fixed in cold methanol/acetone (50%/50% v/v) for 1 min. Blocking step was performed using normal goat serum (10% (v/v), Sigma-Aldrich). Then the cells were incubated for 1 h with a Rabbit anti-ZO1 or Rabbit anti-occludin (1/200^e^ Life Technologies, Carlsbad, CA, USA). After washing, the cells were stained with a secondary antibody (Alexa Fluor 568 anti-rabbit or Alexa Fluor 488, 1/200^e^, Molecular Probes) for 1 h in the dark at room temperature. In each immunofluorescence experiment, an isotype-matched IgG control was used. Cells were mounted using mowiol (Sigma-Aldrich, Saint Quentin Fallavier, France) containing an antifading agent (dabco, Sigma-Aldrich). Nuclei were stained with Hoechst 33358 and then examined with a Leica DMR fluorescence microscope (Wetzlar, Germany). In the last case, images were collected using a CoolSNAP RS Photometrics camera (Leica Microsystems) and were processed using Adobe Photoshop software 5.5 (Adobe Systems).

### 3.8. MTT Tetrazolium Viability Assay 

The MTT (3-(4,5-dimethylthiazol-2-yl)-2,5-diphenyltetrazolium bromide) tetrazolium reduction assay was used with the manufacturer recommendations. After the experiment, Caco-2 cells were incubated with 100 µL of diluted MTT tetrazolium solution during one hour at 37 °C. The quantity of converted MTT into formazan was measured by recording changes in absorbance at 570 nm using a plate reading spectrophotometer (BioTek, H1, Vermont, Winooski) and compared with the control condition after cell solubilization with DMSO.

### 3.9. Quenching Test

Several dilutions of R123 or CMFDA were diluted in RH buffer with 50 µM of test solution and were quantified by fluorescence spectrophotometry (BioTek, H1). The equation of obtained curve was compared with the equation of fluorescent probes alone and confirmed that none of them showed any quenching effect on the fluorescence of probe (Figure S1).

### 3.10. Statistical Analysis

Descriptive statistics (n, means, s.d., s.e.m) and statistical analyses were performed using the Prism 5.0 software. The non-parametric Mann–Whitney Student′s t-test was used with confidence interval of 95%.

## 4. Conclusions

According to the major roles of efflux transporters in drug resistance of tumor cells, but also to their important role in all major physiological barriers (intestine, blood–brain barrier, blood–placenta barrier) as well as in metabolic organs (liver, kidney), it remains essential to explore both substrate and inhibitor properties of newly developed drugs. In addition, these data are compulsory to predict putative drug–drug interactions. There are numerous reported cases where co-administration of a P-gp inhibitor with a P-gp substrate considerably increased the blood levels of the latter, leading to unexpected and serious side effects. Classical examples are drug–drug interactions with digoxin (quinidine), loperamide (ritonavir), saquinavir (tipranavir) or topotecan (elacridar) [24]. The present results suggest that this pump out system based on the use of Caco-2 cells can generate very quickly and, for a very low price, some data relative to P-gp/BCRP and MRPs substrates and inhibitors as well drug–drug interaction mediated through these efflux pump. Loading Caco-2 cells with CMFDA or R213, followed by the measure of efflux of these fluorescent probes, provide a good platform for rapid, and easy to interpret P-gp/BCRP and MRPs interaction studies. In the present study, a rapid fluorescence based microplate screening assay has been introduced to determine drug interactions with P-gp/BCRP and MRPs. However, it should be kept in mind that it is does not distinguish between transported substrates and compounds inhibiting only efflux pump activity. Nevertheless, it provides a useful and fast tool in early compound profiling during drug discovery, and also offers quick feedback regarding the drug design.

In summary, this method to evaluate the drug interaction with major efflux transporters used was shown to provide a rapid and cost-effective strategy. It might be used as a first step in a tiered approach to screen high number of new molecular entities for their possible interactions with efflux pumps. Then, the selected compounds with no efflux pumps interaction properties might be tested in the traditional bi-directional transport assays across polarized cell monolayers model to generate more data on transcellular traffic or interaction with other cellular components or receptors.

## Figures and Tables

**Table 1:** Summary results of K_out_ values of selected test compounds in Caco-2. K_out_ rhodamine123 was 100.0 ± 8.1 (*n* = 34) and 100 ± 12.1 (*n* = 39) for 96- and 384-well plates. K_out_ CMFDA was 100.0 ± 7.6 (*n* = 34) and 100 ± 21.7 (*n* = 39) for 96- and 384-well plates.

			96 Well Plate Format				384 Well Plate Format				Correlation 96/384 Well Plate Format					
		Working Conc.	*k_out_* Rho 123		*k_out_* CMFDA		*k_out_* Rho 123		*k_out_* CMFDA		*Ratio K_out_*		Transporter Interaction			
			pmol.min^−1^.mg^−1^ Protein				pmol.min^−1^.mg^−1^ Protein				96/384 Wells		96 Wells		384 Wells	
Compounds		µM	*n*	*mean*	*n*	*mean*	*n*	*mean*	*n*	*mean*	P-gp/BCRP	MRPs	P-gp/BCRP	MRPs	P-gp/BCRP	MRPs
**1**	**verapamil**	50	34	33.9 ± 7.0	8	72.4 ± 6.6	16	39.3 ± 6.8	43	67.8 ± 16.5	0.86	1.07	+	+	+	+
**2**	**elacridar**	10	29	34.6 ± 8.9	8	71.8 ± 6.6	16	42.5 ± 9.0	44	56.4 ± 14.3	0.81	1.27	+	+	+	+
**3**	**MK571**	50	24	70.9 ± 10.6	24	25.0 ± 6.6	16	59.5 ± 9.9	44	15.7 ± 5.8	1.19	1.59	+	+	+	+
**4**	**diazepam**	50	29	115.2 ± 18.9	8	89.2 ± 8.6	16	101.7 ± 14.6	44	95.1 ± 14.1	1.13	0.94	−	−	−	−
**5**	**acetanomiphen**	50	8	113.7 ± 12.6	8	90.6 ± 6.8	16	105.4 ± 8.2	16	119.1 ± 29.1	1.08	0.76	−	−	−	−
**6**	**amiodarone**	50	8	91.1 ± 10.2	8	83.7 ± 12.7	16	74.5 ± 12.4	16	106.5 ± 17.6	1.22	0.79	−	−	+	−
**7**	**cyclosporinA**	10	8	39.6 ± 9.9	8	53.1 ± 7.6	16	46.4 ± 8.1	16	42.2 ± 10.1	0.85	1.26	+	+	+	+
**8**	**diltiazem**	50	8	51.7 ± 5.9	8	96.4 ± 8.7	16	56.9 ± 7.1	16	87.6 ± 24.1	0.91	1.10	+	−	+	−
**9**	**diclofenac**	50	8	117.3 ± 15.6	8	84.1 ± 7.5	16	91.5 ± 17.4	16	118.7 ± 18.0	1.28	0.71	−	−	−	−
**10**	**loperamide**	50	8	42.0 ± 4.9	8	72.9 ± 4.0	16	41.6 ± 6.2	16	61.2± 16.2	1.01	1.19	+	+	+	+
**11**	**doxorubicin**	20	8	66.7 ± 11.0	8	66.6 ± 5.6	16	64.7 ± 6.8	16	81.3 ± 16.6	1.03	0.82	+	+	+	−
**12**	**phenobarbital**	50	8	107.1 ± 10.0	16	96.6 ± 11.6	16	98.5 ± 7.4	16	94.2 ± 22.4	1.09	1.03	−	−	−	−
**13**	**quinidine**	50	8	36.4 ± 4.2	8	77.6 ± 8.9	16	45.8 ± 6.7	32	84.7 ± 16.9	0.79	0.92	+	+/−	+	−
**14**	**rifampicin**	50	8	66.8 ± 4.4	8	75.5 ± 7.9	16	59.5 ± 8.0	16	76.3 ± 21.5	1.12	0.99	+	+/−	+	+/−
**15**	**vinblastine**	50	8	57.6 ± 3.8	8	77.6 ± 10.6	16	57.9 ± 7.6	32	81.9 ± 17.5	0.99	0.95	+	+/−	+	−
**16**	**tocopherol**	10	8	33.7 ± 2.7	8	88.4 ± 6.9	16	44.6 ± 6.2	32	88.8 ± 15.33	0.76	1.00	+	−	+	−

**Table 2:** From left to right: cDNA targeted for amplification, Forward (F), Reverse (R) primer designation, 5′ to 3′ primer sequence, accession numbers of cDNA from NCBI database.

mRNA	F/R	Sequences	Accession Number
*ABCB1*	F	5′-TCTGCTGTGGAAAAATTACA-3′	NM_013850.1
	R	5′-GACAGCCACTAGGATGAAGA-3′	
*ABCG2*	F	5′-CAAGCATCTTCAGTTCATCAGC-3′	NM_013454.3
	R	5′-GAGTGTAGCAGGGACCACATAA-3′	
*ABCC2*	F	5′-CTCCCAAGTCACACAAGAACTG-3′	NM_009696.3
	R	5′-TCCTCCAGCTCCTTTTTGTAAG-3′	
*ACTIN*	F	5′-CTGAGGACCTTCCGCAAGATGT-3′	NM_138955
	R	5′-GCTTCAGGTTGGCAGAGACCAT-3′	

## Supplementary Materials

The following are available online at https://www.mdpi.com/1422-0067/20/22/5529/s1.

## References

[1] Pedersen J.M., Matsson P., Bergström C.A.S., Norinder U., Hoogstraate J., Artursson P. (2008). Prediction and Identification of Drug Interactions with the Human ATP-Binding Cassette Transporter Multidrug-Resistance Associated Protein 2 (MRP2; ABCC2). J. Med. Chem..

[2] Matsson P., Pedersen J.M., Norinder U., Bergström C.A.S., Artursson P. (2009). Identification of Novel Specific and General Inhibitors of the Three Major Human ATP-Binding Cassette Transporters P-gp, BCRP and MRP2 Among Registered Drugs. Pharm. Res..

[3] Poirier A., Cascais A.-C., Bader U., Portmann R., Brun M.-E., Walter I., Hillebrecht A., Ullah M., Funk C. (2014). Calibration of In Vitro Multidrug Resistance Protein 1 Substrate and Inhibition Assays as a Basis to Support the Prediction of Clinically Relevant Interactions In Vivo. Drug Metab. Dispos..

[4] Hubatsch I., Ragnarsson E.G.E., Artursson P. (2007). Determination of drug permeability and prediction of drug absorption in Caco-2 monolayers. Nat. Protoc..

[5] Sevin E., Dehouck L., Fabulas-da Costa A., Cecchelli R., Dehouck M.P., Lundquist S., Culot M. (2013). Accelerated Caco-2 cell permeability model for drug discovery. J. Pharmacol. Toxicol. Methods.

[6] Brück S., Strohmeier J., Busch D., Drozdzik M., Oswald S. (2017). Caco-2 cells - expression, regulation and function of drug transporters compared with human jejunal tissue. Biopharm. Drug Dispos..

[7] Gameiro M., Silva R., Rocha-Pereira C., Carmo H., Carvalho F., Bastos M.D.L., Remião F. (2017). Cellular Models and In Vitro Assays for the Screening of modulators of P-gp, MRP1 and BCRP. Molecules.

[8] Kerns E.H., Di L., Petusky S., Farris M., Ley R., Jupp P. (2004). Combined application of parallel artificial membrane permeability assay and Caco-2 permeability assays in drug discovery. J. Pharm. Sci..

[9] Balimane P.V, Patel K., Marino A., Chong S. (2004). Utility of 96 well Caco-2 cell system for increased throughput of P-gp screening in drug discovery. Eur. J. Pharm. Biopharm..

[10] Hellinger E., Bakk M.L., Pócza P., Tihanyi K., Vastag M. (2010). Drug penetration model of vinblastine-treated Caco-2 cultures. Eur. J. Pharm. Sci. Off. J. Eur. Fed. Pharm. Sci..

[11] Anderle P., Niederer E., Rubas W., Hilgendorf C., Spahn-Langguth H., Wunderli-Allenspach H., Merkle H.P., Langguth P. (1998). P-Glycoprotein (P-gp) mediated efflux in Caco-2 cell monolayers: the influence of culturing conditions and drug exposure on P-gp expression levels. J. Pharm. Sci..

[12] Hayeshi R., Hilgendorf C., Artursson P., Augustijns P., Brodin B., Dehertogh P., Fisher K., Fossati L., Hovenkamp E., Korjamo T. (2008). Comparison of drug transporter gene expression and functionality in Caco-2 cells from 10 different laboratories. Eur. J. Pharm. Sci..

[13] Young L., Sung J., Stacey G., Masters J.R. (2010). Detection of Mycoplasma in cell cultures. Nat. Protoc..

[14] Pamies D., Bal-Price A., Simeonov A., Tagle D., Allen D., Gerhold D., Yin D., Pistollato F., Inutsuka T., Sullivan K. (2016). Good Cell Culture Practice for stem cells and stem-cell-derived models. ALTEX.

[15] Yamashita S., Konishi K., Yamazaki Y., Taki Y., Sakane T., Sezaki H., Furuyama Y. (2002). New and better protocols for a short-term Caco-2 cell culture system. J. Pharm. Sci..

[16] Balimane P. V, Chong S. (2005). Cell culture-based models for intestinal permeability: a critique. Drug Discov. Today.

[17] Forster S., Thumser A.E., Hood S.R., Plant N. (2012). Characterization of Rhodamine-123 as a Tracer Dye for Use In In vitro Drug Transport Assays. PLoS ONE.

[18] Yamaguchi S., Zhao Y.L., Nadai M., Yoshizumi H., Cen X., Torita S., Takagi K., Takagi K., Hasegawa T. (2006). Involvement of the drug transporters p glycoprotein and multidrug resistance-associated protein Mrp2 in telithromycin transport. Antimicrob. Agents Chemother..

[19] Bui K., She F., Zhou D., Butler K., Al-Huniti N., Sostek M. (2016). The effect of quinidine, a strong P-glycoprotein inhibitor, on the pharmacokinetics and central nervous system distribution of naloxegol. J. Clin. Pharmacol..

[20] Lebedeva I. V, Pande P., Patton W.F. (2011). Sensitive and specific fluorescent probes for functional analysis of the three major types of mammalian ABC transporters. PLoS ONE.

[21] Nakanishi T., Shibue Y., Fukuyama Y., Yoshida K., Fukuda H., Shirasaka Y., Tamai I. (2011). Quantitative time-lapse imaging-based analysis of drug-drug interaction mediated by hepatobiliary transporter, multidrug resistance-associated protein 2, in sandwich-cultured rat hepatocytes. Drug Metab. Dispos..

[22] Bakos É., Homolya L. (2007). Portrait of multifaceted transporter, the multidrug resistance-associated protein 1 (MRP1/ABCC1). Pflügers Arch. Eur. J. Physiol..

[23] Yang C., Wu T., Qi Y., Zhang Z. (2018). Recent Advances in the Application of Vitamin E TPGS for Drug Delivery. Theranostics.

[24] Montanari F., Ecker G.F. (2015). Prediction of drug–ABC-transporter interaction — Recent advances and future challenges. Adv. Drug Deliv. Rev..

[25] Schinkel A.H., Jonker J.W. (2003). Mammalian drug efflux transporters of the ATP binding cassette (ABC) family: an overview. Adv. Drug Deliv. Rev..

[26] Mao Q., Unadkat J.D. (2005). Role of the breast cancer resistance protein (ABCG2) in drug transport. AAPS J..

