# Supplementary material for: A Miniaturized Pump Out Method for Characterizing Molecule Interaction with ABC Transporters
Source: Int J Mol Sci. 2019 Nov 6;20(22):5529. doi: 10.3390/ijms20225529 (PMC6888615; doi:10.3390/ijms20225529)
Supplement: Supplementary file 1 [file ijms-20-05529-s001.zip › Supplementary data.pdf]

Supplementary data S1

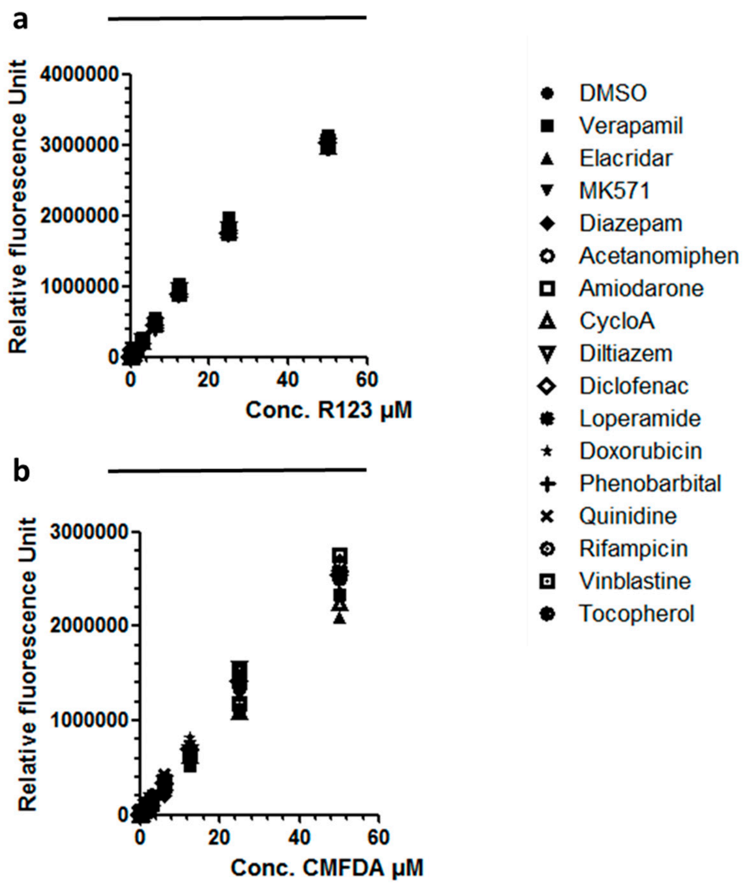

S1: Several dilutions of R123 (a) or CMFDA (b) were diluted in RH buffer with 50 µM of test solution. R123 and CMFDA were quantified by fluorescence spectrophotometry (BioTek, H1). The equation of obtained curve was compared with the equation of fluorescent probes alone and confirmed that none of them showed any quenching effect on the fluorescence of probe.

Supplementary data S2

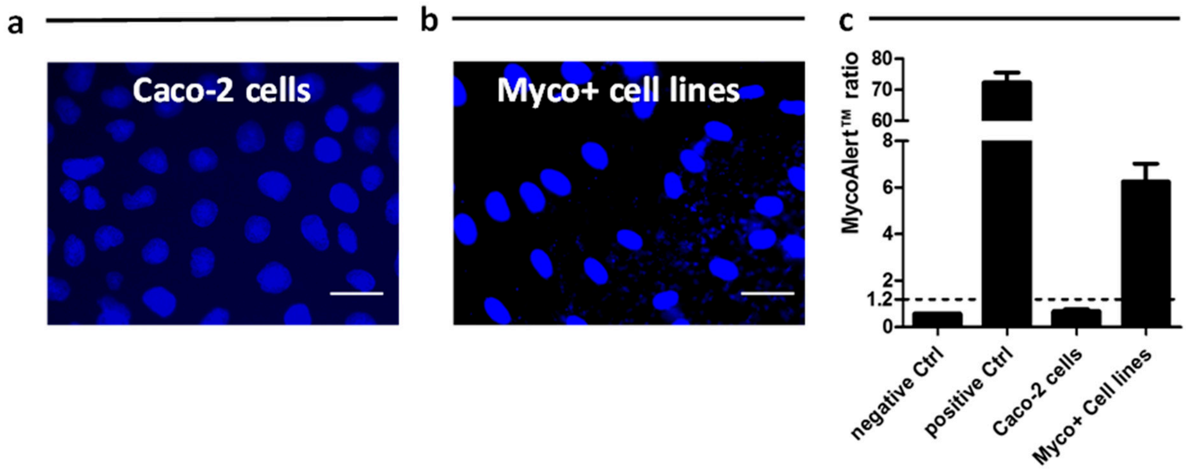

S2: Mycoplasma detection in Caco-2 cell cultures. Nuclei staining of a) Caco-2 cells or b) cell lines contaminated with mycoplasma, with Hoescht 33342. c) Mycoplasma detection with MycoAlert™ detection Kit from Lonza™

Supplementary data S3

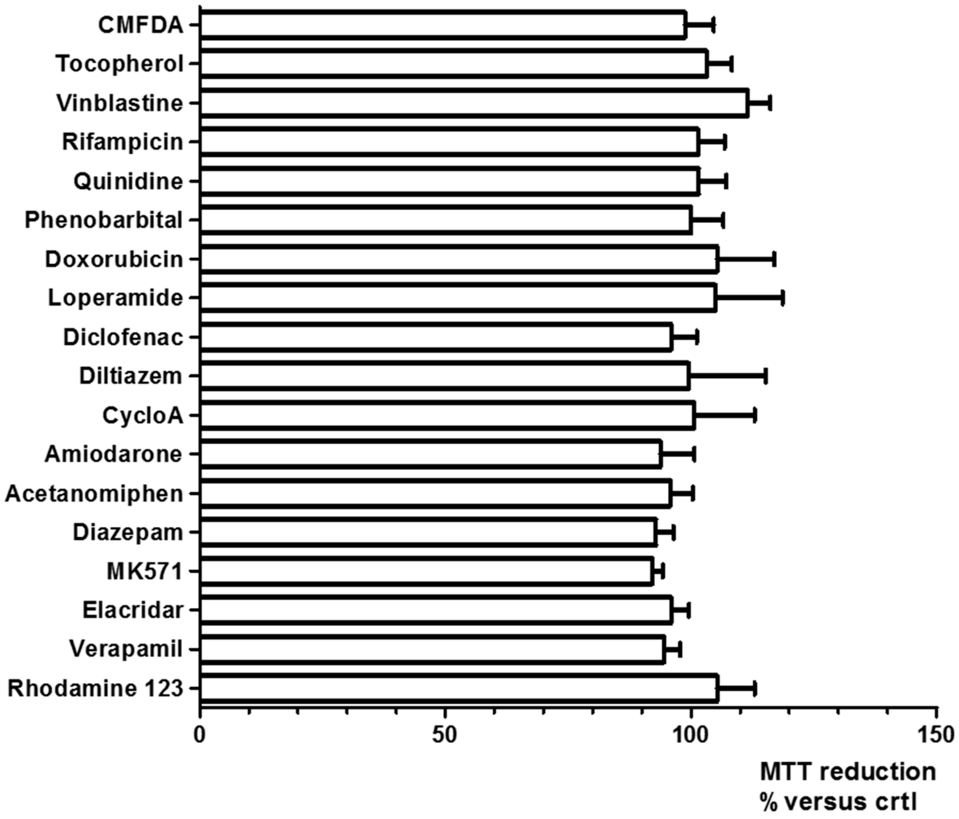

S3: The quantity of converted MTT into formazan was measured by recording changes in absorbance at 570 nm and compared with the control condition after cell solubilisation with DMSO. No cellular toxicity was in the presence of tested compounds.
