# Supplementary material for: A Miniaturized Pump Out Method for Characterizing Molecule Interaction with ABC Transporters
Source: Int J Mol Sci. 2019 Nov 6;20(22):5529. doi: 10.3390/ijms20225529 (PMC6888615; doi:10.3390/ijms20225529)
Supplement: Supplementary file 1 [file ijms-20-05529-s001.zip › Table 1.pdf]

	Working Conc. *Conc.*	96 well plate format				384 well plate format				Correlation 96 / 384 well plate format					
		k_{out} Rho 123 (pmol.min^{-1}.mg^{-1} protein)		k_{out} CMFDA		k_{out} Rho 123 (pmol.min^{-1}.mg^{-1} protein)		k_{out} CMFDA		***Ratio K_{out}*** 96 / 384 wells		Transporter Interaction 96 wells		Transporter Interaction 384 wells	
**Compounds**	*µM*	*n*	*mean*	*n*	*mean*	*n*	*mean*	*n*	*mean*	P-gp/BCRP	MRPs	P-gp/BCRP	MRPs	P-gp/BCRP	MRPs
**1 verapamil**	50	34	33.9 ± 7.0	8	72.4 ± 6.6	16	39.3 ± 6.8	43	67.8 ± 16.5	0.86	1.07	+	+	+	+
**2 elacridar**	10	29	34.6 ± 8.9	8	71.8 ± 6.6	16	42.5 ± 9.0	44	56.4 ± 14.3	0.81	1.27	+	+	+	+
**3 MK571**	50	24	70.9 ± 10.6	24	25.0 ± 6.6	16	59.5 ± 9.9	44	15.7 ± 5.8	1.19	1.59	+	+	+	+
**4 diazepam**	50	29	115.2 ± 18.9	8	89.2 ± 8.6	16	101.7 ± 14.6	44	95.1 ± 14.1	1.13	0.94	-	-	-	-
**5 acetanomiphen**	50	8	113.7 ± 12.6	8	90.6 ± 6.8	16	105.4 ± 8.2	16	119.1 ± 29.1	1.08	0.76	-	-	-	-
**6 amiodarone**	50	8	91.1 ± 10.2	8	83.7 ± 12.7	16	74.5 ± 12.4	16	106.5 ± 17.6	1.22	0.79	-	-	+	-
**7 cyclosporinA**	10	8	39.6 ± 9.9	8	53.1 ± 7.6	16	46.4 ± 8.1	16	42.2 ± 10.1	0.85	1.26	+	+	+	+
**8 diltiazem**	50	8	51.7 ± 5.9	8	96.4 ± 8.7	16	56.9 ± 7.1	16	87.6 ± 24.1	0.91	1.10	+	-	+	-
**9 diclofenac**	50	8	117.3 ± 15.6	8	84.1 ± 7.5	16	91.5 ± 17.4	16	118.7 ± 18.0	1.28	0.71	-	-	-	-
**10 loperamide**	50	8	42.0 ± 4.9	8	72.9 ± 4.0	16	41.6 ± 6.2	16	61.2± 16.2	1.01	1.19	+	+	+	+
**11 doxorubicin**	20	8	66.7 ± 11.0	8	66.6 ± 5.6	16	64.7 ± 6.8	16	81.3 ± 16.6	1.03	0.82	+	+	+	-
**12 phenobarbital**	50	8	107.1 ± 10.0	16	96.6 ± 11.6	16	98.5 ± 7.4	16	94.2 ± 22.4	1.09	1.03	-	-	-	-
**13 quinidine**	50	8	36.4 ± 4.2	8	77.6 ± 8.9	16	45.8 ± 6.7	32	84.7 ± 16.9	0.79	0.92	+	+/-	+	-
**14 rifampicin**	50	8	66.8 ± 4.4	8	75.5 ± 7.9	16	59.5 ± 8.0	16	76.3 ± 21.5	1.12	0.99	+	+/-	+	+/-
**15 vinblastine**	50	8	57.6 ± 3.8	8	77.6 ± 10.6	16	57.9 ± 7.6	32	81.9 ± 17.5	0.99	0.95	+	+/-	+	-
**16 tocopherol**	10	8	33.7 ± 2.7	8	88.4 ± 6.9	16	44.6 ± 6.2	32	88.8 ± 15.33	0.76	1.00	+	-	+	-
